# Understanding the impact of COVID-19 pandemic on health-related quality of life amongst Iranian patients with beta thalassemia major: a grounded theory

**DOI:** 10.1017/S146342362100013X

**Published:** 2021-11-10

**Authors:** Mahdieh Arian, Mojtaba Vaismoradi, Zahra Badiee, Mohsen Soleimani

**Affiliations:** 1 Student Research Committee, Faculty of Nursing and Midwifery, Semnan University of Medical Sciences, Semnan, Iran; 2 Faculty of Nursing and Health Sciences, Nord University, Bodø, Norway; 3 Department of Pediatrics, Faculty of Medicine, Mashhad University of Medical Sciences, Mashhad, Iran; 4 Nursing Care Research Center, Faculty of Nursing and Midwifery, Semnan University of Medical Sciences, Semnan, Iran

**Keywords:** coronavirus, grounded theory, health care, quality of life, beta thalassemia major

## Abstract

**Background:**

The coronavirus disease 2019 (COVID-19) pandemic and the resulting measures can impact daily life and healthcare management amongst patients with beta thalassemia major.

**Methods:**

The Corbin and Strauss method of grounded theory was used to explore the impact of the COVID-19 pandemic on health-related quality of life (HRQoL) amongst Iranian patients with beta thalassemia major. Semi-structured interviews with 16 patients with thalassemia major in the eastern of Iran were performed. Data collection was conducted from 19 September through 18 November 2020. Collected data were recorded, transcribed, and coded to develop themes and subthemes. Paradigm components were sought to find out what happened to these patients and explore the process and events.

**Results:**

Insights from these interviews led to five major themes: ‘changing physical health’, ‘emotional and psychological reactions’, ‘changing the nature of relationships and the scope of social support’, ‘metamorphosis of ongoing healthcare, and ‘functionality and adaptation to new realities.’ The emerging core concept was labelled: ‘maintaining well-being balance.’ The COVID-19 pandemic disturbed the balance of life and health of the patients. Multiple strategies to maintain balance and reduce the negative effects of the COVID-19 pandemic on HRQoL were used by the patients, the healthcare team, and support systems.

**Conclusions:**

Due to the fear of COVID-19, the patients with beta thalassemia were less likely to contact healthcare professionals. They considered postponing blood transfusion and abandoned evaluating disease complications. Reduced access to the healthcare system and shifting resources from existing programmes to COVID-19 by the healthcare system were incompatible policies. These policies and strategies had strong and negative effects on the physical domain of HRQoL. The patients experienced a deterioration of emotional functioning. They reported a strong reduction in social functioning and felt lonely. Online interventions supporting mental health and social interactions and telemedicine can help during the times of social distancing and lockdowns.

## Introduction

In late December 2019, an ongoing outbreak of severe acute respiratory syndrome coronaviruses 2 (SARS-CoV-2) infection, which was termed as coronavirus disease 2019 (COVID-19), was reported in Wuhan, China (Zhu *et al.*, 2020). At the end of February 2020, a new era began: the pandemic of the novel coronavirus, which was found to be sufficiently divergent from severe acute respiratory syndrome coronavirus and to be considered a new human-infecting beta coronavirus (Lu *et al.*, 2020) and completely changed the human life. A total of 98,925,221 have been reported globally, and 1 379 286 confirmed cases have been documented in Iran until 26 January 2021. The death toll from the COVID-19 outbreak has been 2 127 294 worldwide, and 57 481 in Iran (WHO, 2021). Patients with thalassemia major, especially young adults, suffer a chronic condition that is associated with several comorbidities linked to the underlying disease and complications of chronic blood transfusions, including cardiac problems, pulmonary hypertension, and diabetes. Also, there could be an increased risk of more severe COVID-19 disease in these patients (Motta *et al.*, 2020). Nonetheless, outcomes recently reported by a study in Iran showed a low number of β-thalassemia major patients with COVID-19, and patients mostly developed a mild to moderate disease and recovered. It is noted that having multiple comorbidities predispose these patients to severe complications of COVID-19 and causes a significantly higher mortality rate compared to the general infected population (Karimi *et al.*, 2020). Other outcomes were recently reported by a small cohort of Italian patients followed in the northern part of Italy, where the pandemic has been the most widespread, indicating the most relatively mild to moderate COVID-19 disease experienced by patients. The number of infected thalassemia major patients was less than expected, maybe due to earlier and more alert self-isolation compared to the total population (Motta *et al.*, 2020). Just as healthy patients have experienced a life change due to quarantine, so have patients with beta thalassemia major.

During the first 20 days of the pandemic, 7161 cases of COVID-19 were reported in Iran, for a cumulated rate of 8.9 cases/100,000 population, reaching up to 1234 cases during 6 March 2020. All provinces have been affected, and rates ranged from 0.8 in Bushehr province to 61.8 cases/100,000 population in Qom province. COVID-19 arrived in Iran from China and the first 2 cases were found in Qom. Most cases have been reported in Tehran, 1945 people, followed by Qom, 712 people, and Mazandaran that has the highest number of patients with beta thalassemia major in Iran with 633 people. Qom has a border with Markazi and Semnan provinces with 27.2–34.9 cases/100,000 population (eAppendix 1 in the Supplement) (Arab-Mazar *et al.*, 2020). After that, the same figure was seen in other cities including Mashhad as the capital city of Khorasan Razavi with 3.1 cases/100,000 population. Mashhad is the second largest religious city in the world attracting more than 20 million tourists and pilgrims every year, many of whom come to pay homage to the Imam Reza shrine (the eighth Shi’ite Imam) (Arab-Mazar *et al.*, 2020).

There are severe limitations in the Iranian public health system’s medical supplies to deal with the current outbreak of COVID-19. Hospitals have virtually stopped their non-emergency and specialty activities (Arab-Mazar *et al.*, 2020). Patients with thalassemia major are very sensitive and need regular blood transfusions, IV chelation therapy, periodic screenings of thalassemia complications, and psychological counselling. It seems that the government healthcare system should not forget patients with thalassemia major. Before the COVID-19 emergency, one of the most relevant activities of the Iranian healthcare system was the development of strategies for regular periodic screening procedures and avoiding long waiting lists for them in hospitals or clinics, as well as the implementation of programmes for improving health-related quality of life (HRQoL) in these patients. Since the coronavirus pandemic broke out, all public and private hospitals in Iran changed their focus to COVID-19 patients with severe breathing symptoms, as no therapeutic regimen has yet been proven effective for the treatment of COVID-19. Simultaneously, the considerable medical burden of the COVID-19 pandemic with the unprecedented extra load of the healthcare systems across the world and the obvious diversion of medical resources from other diseases to COVID-19 may jeopardise the already demanding and often suboptimal care of patients with beta thalassemia (Farmakis *et al.*, 2020). One of the major fears of most healthcare professionals, governments, and patients is the heavy impact of COVID-19 on healthcare delivery. There is a risk that patients with chronic diseases may not receive treatment on time due to the general encouragement to stay home to prevent the extension of the coronavirus, healthcare staff shortages, and economic crisis.

The complications of chronic diseases on HRQoL is considered important (Lemon *et al.*, 2004). HRQoL is a comprehensive and multidimensional concept and has been defined as physical, role functioning, social, and psychological aspects of well-being affected by the disease and its treatment (Seedhouse, 1986; de Wit and Hajos, 2013). During the pandemic period, patients with thalassemia major are faced with a serious dilemma, since staying at home and postponing blood transfusions or not receiving chelators on time can increase the risk of severe anaemia and iron overload. Also, while visiting the hospital for screenings, thalassemia complications can increase the risk of infection by COVID-19 besides mental and emotional problems caused by staying at home (Eleftheriou *et al.*, 2020).

These patients stand out in the pandemic situation, because of their need to frequent visits to healthcare facilities for blood transfusions. Their conditions make the need for protection measures imperative as hospital environments may be regarded as ‘hotspots’ for viral transmission. According to Thalassaemia International Federation (TIF), the pandemic has secondary consequences, such as blood shortages, medication shortages, and reduced access to specialised care (Farmakis *et al.*, 2020). TIF closely evaluates the impact of the COVID-19 pandemic on HRQoL and compiles the emerging scientific evidence relevant to the care of patients with haemoglobinopathies. This condition is particularly pivotal for patients living in developing or low-income countries, where disease-specific treatment is lacking (Farmakis *et al.*, 2020).

This study aimed to understand the impact of the COVID-19 pandemic on HRQoL amongst Iranian patients with beta thalassemia major. Given the changes in the life experiences of these patients due to the COVID-19 pandemic, qualitative research could better identify the altered dimensions of HRQoL.

## Methods

### Objectives

This study aimed to understand the impact of the COVID-19 pandemic on HRQoL in terms of all physical, psychological, social, and functional aspects of well-being affected by the disease and the treatment process amongst Iranian patients with beta thalassemia major.

### Design

This qualitative research was performed based on the approach of grounded theory suggested by (Corbin and Strauss, 2015). It is used to deeply explore the complex social phenomena that cannot be deeply understood by using procedural and rigid quantitative methods. The COVID-19 pandemic has manifested itself in different forms and intensities in different countries due to different public policies and different cultures, which play a fundamental role in people’s behaviours. Therefore, the care context is very important in understanding and interpreting the findings of grounded theory research (Corbin and Strauss, 2015). This article adheres to the Consolidated Criteria for Reporting Qualitative Research (COREQ) reporting guideline (eAppendix 2 in the Supplement) (Tong *et al.*, 2007). The key elements of grounded theory used in this research have been summarised in Table 1.

**Table 1.** Summary of data collection and analysis methods (Corbin and Strauss, 2015)

Method	Description
Theoretical sampling	It maximises opportunities for discovering variations in concepts and is used to densify themes/categories in terms of their properties and dimensions.
Constant comparison	Process of comparing data in order to emerge categories.
Open coding and identification of concepts	Labelling and conceptualising a large amount of raw interview data sentence by sentence; breaking data apart and delineating concepts to stand for interpreted meaning of raw data.
Development of concepts in terms of dimensions and characteristics	The process of acquiring sufficient data to develop each category or theme fully in terms of its properties and dimensions and to account for variation.
Analysing data for context	Analysis of data for context helps identify the range of possible conditions that are operating in any situation and in turn the range of consequences that result from action–interaction.
Bringing process into the analysis	Bringing process into the analysis helps represent the rhythm as well as the changing and repetitive forms of action–interaction plus the pauses and interruptions that occur when persons act and interact for the purpose of reaching a goal or solving a problem.
Integrating categories	Linking categorises around a core category and refining and trimming the theory.
Analytic memos	Notes are written by the researcher for linking and explaining concepts and emerging categories.
Theoretical sensitivity	Having insights as well as being tuned into and being able to pick up on relevant issues, events, and happenings during data collection and analysis.
Theoretical saturation	It is reached when no new data are collected to improve the depth of data analysis.

**Figure 1.**
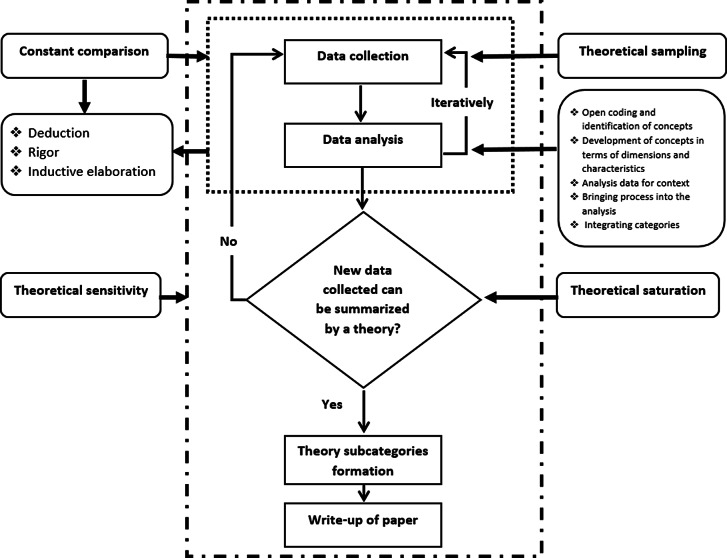
Data collection and analytic methods (Corbin and Strauss, 2015, Dunn *et al.*, 2017, Fu *et al.*, 2019)

These elements included theoretical sampling, constant comparison, open coding and identification of concepts, development of concepts in terms of dimensions and characteristics, analysis data for context, bringing the process into the analysis, integrating categories, writing a memo, theoretical sensitivity, and theoretical saturation, as have been described by (Corbin and Strauss, 2015). The data collection and analytic methods have been described in Figure 1.

### Setting and study population

The participants were chosen using theoretical sampling (Corbin and Strauss, 2015) from the thalassemia association with the consideration of maximum variation in sampling in terms of age, gender, ethnicity, and education level in order to reach greater insights into the study phenomenon for the formulation of the theory. Eligibility criteria were to currently reside in Mashhad city and ability to speak Persian and communicate via mobile phone or email. Using a standardised script, the thalassemia association’s secretary conducted general outreach via telephone calls to patients with thalassemia major to assess them in terms of eligibility and interest in participation in this study. The first author (M.A) then approached voluntary individuals via email or telephone with a standardised script explaining the research aim and method and were invited to be interviewed. The participants were given the option of face-to-face or phone interview. According to (Sturges and Hanrahan, 2004), telephone interviews can produce the same results as face-to-face interviews. Thirteen of the 16 participants chose the phone interview method. Once the participants agreed to participate, the interview was scheduled.

Data about the patient’s age, gender, educational level, and employment history were collected. A total of 16 patients participated in this study (Table 2). Two participants were interviewed twice to remove ambiguities during the data collection. Data collection was conducted from 19 September through 18 November 2020.

**Table 2.** The general characteristics of the patients (*n* = 16)

Participants	Age, years	Gender	Education level	Marital status	COVID-19	Comorbidities
P1	37	Female	College	Single	No	Heart complication, hypocalcaemia, splenectomy
P2	25	Female	High school	Single	No	Heart complication, hypothyroidism
P3	28	Male	College	Single	Mild	Heart complication, diabetes mellitus, splenectomy
P4	37	Male	High school	Married, living with spouse	Mild	Heart complication, hepatitis, splenectomy
P5	29	Male	Master’s degree	Single	No	Heart complication, borderline diabetes
P6	35	Female	College	Married, living with spouse	Mild	Heart complication, hepatitis, splenectomy, borderline diabetes
P7	19	Female	High school	Single	No	Heart complication
P8	24	Female	High school	Single	No	Heart complication, diabetes mellitus
P9	19	Male	High school	Single	No	Heart complication
P10	27	Female	High school	Widowed, living with parents	No	Heart complication, splenectomy
P11	34	Male	High school	Married, living with spouse	No	Heart complication, splenectomy
P12	33	Male	Ph.D. degree	Single	No	Hypocalcaemia, splenectomy
P13	32	Female	High school	Widowed, living with parents	No	Heart complication, hepatitis, splenectomy
P14	26	Male	College	Married, living with spouse	Moderate	Borderline diabetes, hypothyroidism
P15	37	Female	High school	Married, living with spouse	No	Heart complication, diabetes mellitus, splenectomy
P16	29	Male	College	Single	No	Heart complication, splenectomy

COVID-19 = coronavirus disease 2019.

### Interview outline

Semi-structured interviews with open-ended questions were used. Prior to commencing the research, a review of literature was carried out to design the interview guide. Also, two patients were pre-interviewed to check the interview outline. The main interview questions were as follows: How did you feel during the COVID-19 pandemic? How did the COVID-19 pandemic affect your life and your health? How did you respond to this situation? What did you feel or think about COVID-19? Are there additional concerns or issues you would like to talk about? Do you have any questions from me? (eAppendix 3 in the Supplement).

### Data analysis

Immediately following the interview, data transcription was created from audio recordings, which then was uploaded to the MaxQDA 2020 software for data management. Open coding was begun when the interview was still fresh. Following the coding process, the researcher composed a memo describing the interview situation. Using the constant comparison method and theoretical sampling, data collection and data analysis occurred simultaneously. Data gathered through a deductive process was validated, resulting in inductive elaboration (Corbin and Strauss, 2015), by which the resulting theory was grounded in the collected data (Creswell and Poth, 2016).

### Constant comparison

The data analysis aimed at developing a theoretical explanation of how the COVID-19 pandemic impacted on HRQOL amongst Iranian patients with beta thalassemia major. Deduction, validation, and inductive elaboration were used to find a theoretical explanation of the actions and process of HRQOL during the COVID-19 pandemic (Corbin and Strauss, 2015). Through creating a verbatim transcript of the interview following each interview and before coding, the researcher had ample time to reflect on each interview. Also, the analytic memo helped create and link categories/themes, and describe the process (Corbin and Strauss, 2015). The memos as key analytical tools helped with developing categories and themes and were the vital part of the theory creation. Coding was used to focus on actions occurring or being described by the participants using the general question, ‘what is going on here.’ Evaluation codes were used to find the perspectives of patients on facilities, resources, actions, and behavioural strategies (Miles *et al.*, 2019).

### Open coding and identification of concepts/development of concepts in terms of dimensions and characteristics

During open coding and identification of concepts using a line-by-line technique, categories and themes were developed. For the development of concepts in terms of dimensions and characteristics, on the part of the researcher, creative and solid data analysis required astute questioning, a relentless search for answers, active observation, and accurate recall. It is a process of fitting data together, of making the invisible obvious of linking and attributing consequence to the antecedent. It is a process of conjecture and verification, of correction and modification, of suggestion and defence. It is also the process of acquiring sufficient data to develop each category or theme fully in terms of its properties and dimensions and to account for variations. Given the questions of who, what, when, and how their properties or dimensions were found. By constant comparison methods, codes were created on the fly as new concepts emerged (Corbin and Strauss, 2015). As the coding structure began to emerge, subcodes were added to similar concepts. Two lead investigators (M.A. and M.S.) reviewed the 16 transcripts and consolidated the codes into a final codebook (eAppendix 4 in the Supplement). The code cloud has been illustrated in (eAppendix 5 in the Supplement). A total of 335 segments were coded. A total of 143 codes were created, resulting in 5 major themes.

### Analytic and methodological memos and diagrams

Following each interview, a memo was written summarising the interview and commenting on theoretical concepts. Methodological memos were created to clarify methods, direct theoretical sampling approaches, and define the emerging codes’ dimensions and characteristics. Analytic memos helped with expanding theoretical concepts. The memos and coded segments formed the basis for creating a diagram designed to visualise the process, resulting in the formation of the theory (Corbin and Strauss, 2015).

### Analysis data for context/bringing process into the analysis

**Figure 2.**
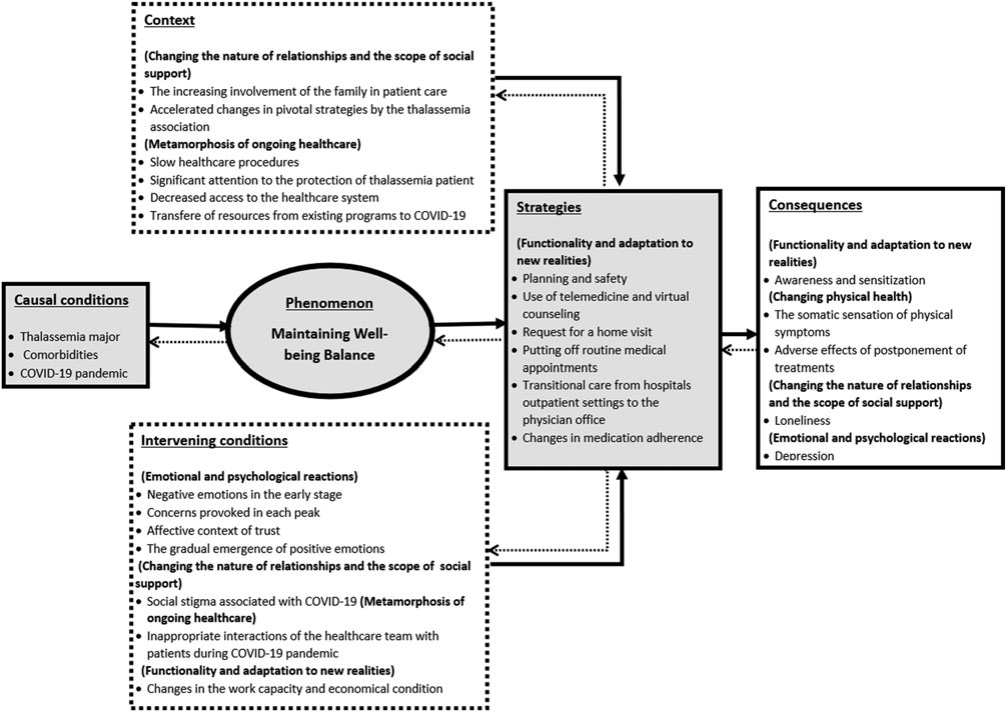
Linking themes developed during the data analysis

Context is a complicated notion. It locates and explains action–interaction within a background of conditions and anticipated consequences. In doing so, it links concepts and enhances a theory’s ability to explain. Bringing process into the analysis helps to represent the rhythm as well as the changing and repetitive forms of action–interaction plus the pauses and interruptions that occur when persons act and interact for the purpose of reaching a goal or solving a problem. Adaptive changes in the flow of action–interaction taken in response to changes in conditions, the changes deemed necessary to achieve desired outcomes or reach a goal. Action–interaction may be strategic, routine, random, novel, automatic, or thoughtful. Analysis data for context and bringing the process into the analysis, linked codes and concepts, gave a form to data, including the variation, complexity, integration, and level of abstraction necessary to go beyond a description of the phenomenon to a theoretical explanation. This led to the development of categories and themes and a description of the core phenomena, actions, and processes, including causal conditions, strategies, intervening conditions, context, and consequences (Corbin and Strauss, 2015). This step included the use of the interviews’ coding segments and memos to determine dimensions, context, interactions, and relationships between the codes. The linking of themes has been illustrated in Figure 2.

### Integrating categories

As the final phase of the data analysis process, a unified theoretical explanation of HRQoL using integrating categories was developed. Theory construction sets grounded theory apart from other qualitative methods by moving beyond the description of who and what to explain why and how (Corbin and Strauss, 2015). Achieving a theoretical integration of the themes and categories requires a description of the properties, dimensions, density, and variation in the data. The participants were frequently asked to go a little deeper if their answers appeared superficial during the interviews. The theory formation included the theoretical integration of key themes. Techniques used to develop the theory consisted of the use of analytic memos and diagrams to further explain the process of HRQoL amongst patients with thalassemia major during the COVID-19 pandemic. The linking of themes from each research question led to developing the HRQoL model.

### Ethical considerations

The study protocol was approved by the ethics committee of the institute, where the corresponding author (M.S.) worked (decree code: IR.SEMUMS.REC.1397.244). The consent form was signed electronically and sent via email. The participants were informed that participation was voluntary and that they could withdraw at any time. It was also emphasised that their privacy would be protected, and their identity would never be revealed. The participants were given an ID number, and the interviews’ transcripts replaced all names with their ID numbers. The participants also were asked to give consent to audio recording of the interviews.

### Rigour

According to Corbin and Strauss (2015), credibility indicates trustworthy and believable findings that reflect the experiences of participants, researchers, and readers about the study phenomenon. Therefore, to improve credibility, the memos were written during sampling, data collection, analysis, and the research team continuously assessed analytical decisions. Discussions were held within the research team to reach a consensus on research steps. The first author (M.A.) was engaged with the patients and attracted their trust to collect in-depth data. She documented her own perspectives to avoid bias during the interpretation of findings. Using the flip-flop technique and waving the red flag, the researchers followed up on negative cases and reduced bias. The researchers guided the development of the conceptual model by asking frequent and specific questions. As member checking, two participating patients assisted with the verification of data reflecting their perspectives.

## Results

The participant’s mean age was 29.4 years and 8 (50%) were women. Also, 4 (25%) reported to be suspected or were confirmed cases of COVID-19 from mild to moderate. The other characteristics of the patients have been shown in Table 2.

**Figure 3.**
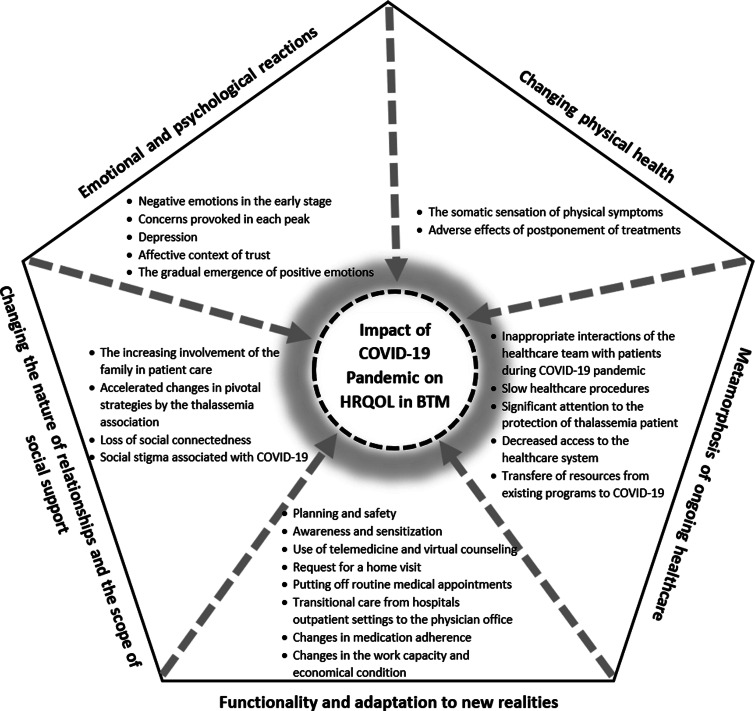
Themes and subthemes developed in this study

The data analysis resulted in developing five major themes consisting of subthemes (Figure 3). The data analysis products have been described using quotations from the participants.

### Theme I: Changing physical health

During the COVID-19 pandemic, the patients mostly could not meet the doctor or attend screening tests due to the fear of COVID-19. Hospitals’ outpatient clinics were temporarily closed during peak periods leading to changes in the treatment process. Emergency departments at public hospitals were allocated to the admission of COVID-19 patients, which led to the postponement of healthcare treatments and exacerbation of physical symptoms amongst the patients.

**P13**: “I referred to the healthcare clinic for blood transfusion 9 days later than the monthly booked time due to fear of getting corona. My haemoglobin level was down, I felt weak and lazy, and had shortness of breath, and forced to spend time in bed.” (Female, 32 years old)

**P1**: “From the beginning of corona, for 8 months ago, I have not taken any blood tests except the blood test; my symptoms show that my blood sugar is high, bit I have not been able to consult with my doctor yet.” (Female, 37 years old)

They believed to be at a higher risk of getting COVID-19 due to splenectomy and related comorbidities.

**P6**: “I have hepatitis, heart disease, and borderline diabetes; my spleen also has been removed, so I got corona earlier than others, but my symptoms were mild. “(Female, 35 years old)

### Theme II: Emotional and psychological reactions

The patients were mentioned to be at a high risk of psychological complications because of the chronic identity of beta thalassemia major. Negative emotions were developed in these patients in the early stage of the COVID-19 pandemic. They panicked because they did not know whether the coronavirus could be transmitted through blood transfusions. Also, changes in the treatment environment and the strange appearance of the healthcare team due to protective equipment led to anxiety. In the early stages of COVID-19, the patients had concerns, because the healthcare system was busy with combating COVID-19, and they anticipated a lack of support for patients with beta thalassemia major by the healthcare system. Their concerns were heightened during the COVID-19-pandemic. They were mostly worried about the reduced blood donation and lack of access to healthcare facilities. Distrust in the healthcare system as an unpleasant feeling was generated because of hearing contradictory news.

**P13**: “I was positive for corona. My blood was transfused in an isolated room. The nurses had difficulties to find someone to take care of me during blood transfusion. The nurse put on a strange dress during the procedure, but my doctor did not visit me that day…. I had worries what would happen to me if I would catch allergic to the transfused blood; I lost my trust in the healthcare team.” (Female, 32 years old)

On the other hand, positive emotions emerged gradually due to the healthcare system’s positive functions, and the patients reached satisfaction and calmness by eliminating their initial concerns.

**P1**: “I feared corona because I thought that I would not be cured given my comorbidities. I heard that my friends with worse physical conditions recovered from corona, so I felt relieved. I thought that if I became infected by corona, the healthcare system is well-equipped, and I will survive.” (Female, 37 years old)

**P16**: “Injecting seasonal flu vaccine for free by the healthcare system alleviated my concern. It showed that thalassemia patients are not neglected.” (Male, 29 years old)

### Theme III: Functionality and adaptation to new realities

The patient’s adherence to infection prevention and control protocols was due to obtaining detailed information on how to prevent the transmission of COVID-19. Because of their physical health condition and weak immune system, they were at a high risk for COVID-19. They minimised the frequency of visits to healthcare facilities to reduce the risk of COVID-19. The use of telemedicine, virtual counselling, or home visit, and visiting the doctor’s office were options to avoid hospital’s outpatient clinics. Postponing blood transfusions, not seeing a doctor, or not following up on screening programmes were inappropriate strategies. COVID-19-pandemic led to a financial burden on the patients because they had to pay for visits to the doctor’s office or home visits during COVID-19 pandemic peaks.

**P8**: “I had a heart complication sometimes ago, and I needed to be hospitalized. My doctor disagreed with admission to the hospital, because of splenectomy, and therefore, my immune system was weak, so he was visiting me at home every day. After that I was recovered, I followed my cardiac rehabilitation program online and sometimes via telephone counselling. The cost of my treatment was very high.” (Female, 24 years old)

The patients needed to quit their jobs to prevent being infected by COVID-19 and experienced changes in adherence to iron chelator therapy.

**P9**: “Due to quarantine and restrictions in the city, I had to go home early at night and inject iron chelators on time.” (Male, 19 years old)

**P1**: “Changes in sleep pattern, caused by quarantine and staying at home made me to feel drowsy or lazy, and injected iron chelators irregularly.” (Female, 37 years old)

### Theme IV: Changing the nature of relationships and the scope of social support

The COVID-19-pandemic had a significant effect on the amount, type, and scope of the patients’ social support. The patients believed that quarantine days increased intra-family communication so that attention to the patients by family members was greater than before. Their parents restricted their freedom for leaving the home during the pandemic. The family members prepared medications or arranged an appointment with the doctor, so the patients did not leave home. Also, their social interactions outside the home were declined, and the patients felt extremely lonely.

**P7**: “Before the corona outbreak, my father did not interfere much in my treatment process, but now he is very attentive to me and prepares my medications, and he even takes me to the doctor’s office and arrange appointments. I sit in the car and he announces when it’s my turn.” (Female, 19 years old)

**P2:** “I have not seen my friends since the association’s entertainment programs were cancelled due to the corona outbreak. I rarely leave the house. I’m not aware of my friends and they are unaware of me.” (Female, 25 years old)

One of the most important supporters for these patients was the thalassemia association. Since the beginning of corona, it changed its strategies and programmes to combat the COVID-19 pandemic and protect beta thalassemia major patients from COVID-19. This association cancelled exercise and entertainment initiatives, provided telephone/online follow-up on patients’ requests, and e-learning for patient education about blood transfusion safety. With the help of donors, this association prepared and distributed health and livelihood packages for improving the patients’ welfare.

**P3**: “The performance of the thalassemia association in this epidemic is very admirable. They cancelled all entertainment programs and delivered healthcare packages, so I did not have to leave home. Through public media channels, they were constantly teaching about COVID-19. Also, any time that I called and asked them to do something, they did it very quickly. Also, they have recently launched online psychological and educational counselling.” (Male, 28 years old)

### Theme V: Metamorphosis of ongoing health care

The effects of changes in healthcare policy during the COVID-19 pandemic on the health and quality of life of the patients were emphasised. Implementation of new healthcare policies in the healthcare system affected the level of access to facilities and equipment.

**P12**: “Two main public hospitals in Mashhad, where most of the famous physicians provide healthcare services to thalassemia patients, are dedicated to admitting patients with corona. Hospital outpatient clinics of these two hospitals during the corona peaks are closed. Other public hospitals don’t admit thalassemia patients without coordination.” (Male, 33 years old)

The patients acknowledged that the importance of diagnosing COVID-19 in the healthcare system increased the triage pre-entry time. Disinfection of the doctor’s office after each visit increased the duration of the healthcare visits, while daily visits were limited. Also, specialised laboratories were very crowded, because the suspected patients were mostly referred for COVID-19 tests, which increased the risk of COVID-19. Healthcare resources such as medical staff, equipment, and facilities were shifted to hospitals involved in COVID-19. Some physicians were infected by a coronavirus and were not available until the end of their quarantine period.

**P4**: “Echocardiograph set was brought into the thalassemia clinic, so we did not have to go to the hospital, but the doctor who performed the echo became ill and no replacement was available to take the role.” (Male, 37 years old)

**P16**: “Corona became a barrier to establishing an MRI ward in the public hospital, given than the hospital and all its wards were reserved for corona. I must travel to Tehran to undergo MRI and measure iron overload in my blood. Unfortunately, a special laboratory in Tehran has announced that it will not admit us during corona peaks.” (Male, 29 years old)

The thalassemia clinic used effective strategies to reduce the transmission of coronavirus to these patients. It was equipped with an isolation room in which patients with COVID-19 performed blood transfusions separately from other patients. Accumulation of the patient’s companions in the clinic was prohibited except for children’s companions. At monthly referrals for visits and blood transfusions, the patients were assessed for COVID-19 symptoms seasonal flu vaccine was available free of charge. Also, specific education and training programmes for nurses and doctors about COVID-19 were available.

## Discussion

This study using a grounded theory design aimed to understand the impact of the COVID-19 pandemic on HRQoL amongst Iranian patients with beta thalassemia major. The paradigm components and the process associated with the patients’ experiences were explored. The emerging core concept was ‘maintaining well-being balance.’ It was found that the COVID-19 pandemic disturbed the balance of life and health of patients with beta thalassemia major. Multiple strategies to maintain this balance and reduce the negative effects of the COVID-19 pandemic on HRQoL were used by the patients, the healthcare team, and support systems. The components of the paradigm have been discussed below.

**The core concept or phenomenon** was labelled: ‘maintaining well-being balance.’ Well-being refers to the quality of psychological, physical, social, and functional wellness. Well-being balance describes a scenario where these elements in a specific context are in the ‘correct proportions’ and patients can maintain them in an ongoing steady state. There are societal, cultural, and medical norms and expectations of what defines reasonable quality in their psychological, physical, and mental wellness. However, each of these patients will also have their own unique perspectives that are manifested in their day-to-day life (Haggett, 2016). Three key principles give relevance and meaning to this definition: well-being balance is subjective, well-being balance is dynamic, and resilience underpins their ability to maintain well-being balance. Well-being balance is subjective because it reflects different individual needs and preferences. What is a need or preference for someone will not be necessarily the same for others. Well-being balance is dynamic because patients and their support systems’ needs and preferences are themselves dynamic and can change. They reflow and flow to reverberate what is happening and essential for them to meet life and health responsibilities. Resilience underpins their ability to maintain a steady state of well-being, because it’s resilience that enables them to regulate, adapt, and reply to ongoing changes in their environment, including those that are unexpected. Resilience is the foundation and allows the patients to respond to changing needs while also, over time, maintain a steady state in their well-being (OECD).

**The causal conditions** include patients with thalassemia major with comorbidities and COVID-19 pandemic. There appeared to be more emotional issues, such as anxiety, but have not experienced a life-threatening event yet caused by the COVID-19 pandemic. The demographic conditions may be important, but in this study, the participants had almost the same conditions in terms of the level of health literacy and economic status.

**Intervening conditions** include the patient’s psychological, social, and functional status, and changes following health policies caused by the COVID-19 pandemic. Psychological status includes negative emotions in the early stage of the COVID-19 pandemic such as fear of blood transfusion; anxiety caused by environmental changes; lack of knowledge and concerns provoked in each peak such as being worried about reduced blood donation and concerns over anticipated lack of thalassemia support by the government and lack of access to facilities. These have impacted their readiness to accept changes and diminished their ability to manage the process of changes and seek for help. It is believed that unbalanced psychological conditions have negative effects on readiness and coping with changes during the COVID-19 pandemic (Vinkers *et al.*, 2020)

According to the patients, the affective context such as trust in the healthcare environment, healthcare workers, organisations, and the government, self-prevention ability, and trust in God in all circumstances played an important role in the patient’s performance to manage the situation. Likewise, if the patient lost trust in the caring context, strategies developed by the healthcare system were considered ineffective, but over time, they perceived the importance of received strategies. Such a trust made the emergence of gradually positive emotions including calmness after notifying the recovery of patients with beta thalassemia major affected by COVID-19, calmness after seasonal influenza vaccination, and satisfaction with multiple social support resources. Trust and confidence are pivotal as is the path out of the current crisis. They shape and are shaped by policy reactions in complicated ways. Trust between patients and the healthcare system is seen as essential to facilitate good healthcare policies. This case has become an eminent dispute during the COVID-19 pandemic. It is believed that trust is associated with a greater adaptation of policy measures. Also, the attitudes of those around patients and public media mediate this relationship. Patients’ trust is increased considerably at the onset of lockdown measures given institutional trust feeding social trust, but direct exposure to COVID-19 reduces trust (Devine *et al.*, 2020).

Also, loss of social connectedness and lack of personal engagements caused by observing physical distancing and self/social isolating during the COVID-19 pandemic diminished the patients’ mood and impacted the process of healthcare follow-up. The current COVID-19 pandemic has provoked social stigma and discriminatory behaviours against anyone who has perceived to have been in contact with COVID-19. The level of stigma associated with COVID-19 amongst patients with thalassemia major is influenced by factors such as it is a new disease and for which there are still many unknowns. Human being is often afraid or confused about the unknown, and it is easy to associate that fear with others. Fear also stimulates harmful stereotypes, labelling, separation, or the experience of the loss of status, because of a perceived link with a disease. Stigma can disturb social integrity and create social isolation, which might contribute to a situation where the virus is more likely to spread. Stigma can drive people to conceal the disease to avoid discrimination, prevent people from inquiring about health care on time, and disappoint them from pursuing healthy behaviours (WHO, 2020).

It seems that during the last few months, as the COVID-19 situation has evolved, the healthcare team has increased their social distance with patients by hiding their faces behind masks, gloves, and imposing personal protective equipment. The patients had the impression that they were spending less time with them and felt coldness and distance. It increases psychosocial distress within their life and impacts their chronic health conditions. Financial difficulties have been provoked by changes in their work capacity and economy during the COVID-19 pandemic, and patients could not afford medical and screening expenses (Pavate, 2020).

**Behavioural strategies** address readiness for change and reduction of concerns following treatments and are used to motivate, engage, and protect the patient. Threat-perceived risk by the patients is the key to benefit from these strategies. There are many functional strategies including planning and safety, telemedicine, virtual counselling, requesting a home visit, putting off routine medical appointments, shifting from hospital outpatient settings to the physician office, and medication adherence. Telemedicine and virtual counselling were important strategies for managing psychological problems during the COVID-19 pandemic via remote consultation. The worst strategy was to abandon routine medical appointments such as deferral blood transfusions and preferred annual screenings for evaluating disease complications that could be dangerous and life-threatening. During the COVID-19 pandemic, most patients shifted their care from the hospital’s outpatient settings to physician offices or requested a home visit, because of the high risk of the transmission of COVID-19. According to the patients, medication adherence was improved by taking an advantage of opportunities during quarantine and also was affected by changes in the sleep pattern or lack of timely preparation of medications. It has been shown that patients with a chronic disease perceive themselves at a high risk for COVID-19 infection and are worried about being infected than healthy people. They experienced the shortages of medications and exhibited adverse lifestyle behaviours leading to declined health (Yan *et al.*, 2020). It is evident that healthy behaviours and strategies can modify and maintain wellness components amongst patients with beta thalassemia major (Biswas *et al.*, 2020). The long-lasting impact of the pandemic on health behaviours, and the possibility of several COVID-19 peaks, emphasise the need for creative and evolving, multilevel approaches to assist these patients to be able to adapt healthy behaviours and prevent disease comorbidities and COVD-19 (Eleftheriou *et al.*, 2020).

**The context** includes the support system of the patient and their access to healthcare facilities. The patients emphasised the role of social and emotional support. The patients mentioned having difficulty processing information and focusing on their self-protection, self-care, visiting a doctor, and following up screening programmes. Once they understood their support system’s actions and strategies, they became relaxed and had a greater ability to focus and learn. Supportive interventions included increasing family involvement in patient care and protection, accelerating changes in pivotal strategies by the thalassemia association, and protection by the healthcare system. However, some healthcare policies had negative consequences for the patient’s HRQoL including slower procedures, decreased access to the healthcare system, and shifting resources from existing programmes to COVID-19.

**The consequences** include awareness and sensitisation that influence on patients’ function, anxiety and managing and resetting conditions, and maintaining life–health balance. Not attending routine medical appointments and non-adherence to medications were negative strategies. On the other hand, the reduction of access to the healthcare system and shifting resources from existing programmes to COVID-19 were incompatible policies used by the healthcare system. These policies and strategies had strong and negative impacts on the physical domain of HRQoL, so the somatic sensation of physical symptoms and postponement of treatments led to an imbalance in life and health. In such a situation, the immune system became weak and predisposed the patients to be infected by COVID-19.

Loneliness is often explained as being without any company or in isolation from the community or society. It is a dark and miserable feeling, and a risk factor for much mental disturbance such as depression, anxiety, adjustment disorder, chronic stress, and insomnia (Wilson *et al.*, 2007). Long periods of isolation in quarantine have detrimental effects on mental well-being (Stickley and Koyanagi, 2016). Social distancing to prevent the spread of infection makes the lonely individual more segregated into his/her own constricted space. Loneliness is also one of the prime indicators of social well-being (Cacioppo and Patrick, 2008). Social isolation leads to chronic loneliness and tedium and if it is long enough, it can have damaging effects on physical and mental well-being. Isolation is combined with mass panic and anxiety, and depression. According to the patients, crisis often affected their minds in crucial ways, increased their anxiety and depression, led to the feeling of unhappiness and unmotivated, and changed their sleep pattern. The cancellation of entertainment programmes of the thalassemia association and loneliness made them depressed. The caring approach in the COVID-19 pandemic such as the lack of communication by the healthcare team or the use of virtual programmes or telemedicine and prolonged cyberspace, increase loneliness and depression, and even can threaten the patient’s life (Banerjee and Rai, 2020).

## Conclusion

Due to the COVID-19 pandemic, patients with thalassemia major are less likely to refer to the healthcare system. They often postpone blood transfusion sessions and the assessment of the complications of the disease. Reduction of access to the healthcare system and shifting resources from healthcare programmes to COVID-19 were inappropriate policies affecting the patients with thalassemia major on their physical and psychological domains of HRQoL. The patients experienced a deterioration in emotional functioning, a sharp reduction in their social functioning, and loneliness. Online interventions supporting mental health and social interaction and telemedicine are needed during the time of social distancing and lockdowns.

### Limitations

This study was conducted in an urban area in the eastern part of Iran. Therefore, the transferability of findings to other cultures and contexts needs further studies.

## Supplementary material

For supplementary material accompanying this paper visit http://dx.doi.org/10.1017/S146342362100013X. click here to view supplementary material
