# Supplementary material for: Understanding the impact of COVID-19 pandemic on health-related quality of life amongst Iranian patients with beta thalassemia major: a grounded theory
Source: Prim Health Care Res Dev. 2021 Nov 10;22:e67. doi: 10.1017/S146342362100013X (PMC8581459; doi:10.1017/S146342362100013X)
Supplement: Supplementary file 1 [file phcsup.zip › S146342362100013Xsup001/S146342362100013Xsup003.docx]

Supplementary Online Content

# Understanding the Impact of COVID-19 Pandemic on Health-Related Quality of Life among Iranian Patients with Beta Thalassemia Major: A Grounded Theory

The authors have provided the following supplementary materials to give readers additional
information about their research.

**eAppendix 1**. The COVID-19 situation in Iran

**eAppendix 2**. The COREQ 32-­‐item checklist

**eAppendix 3**. The interview guide

**eAppendix 4**. The codebook

**eAppendix 5**. The code cloud

**eAppendix 1**. The COVID-19 situation in Iran

| 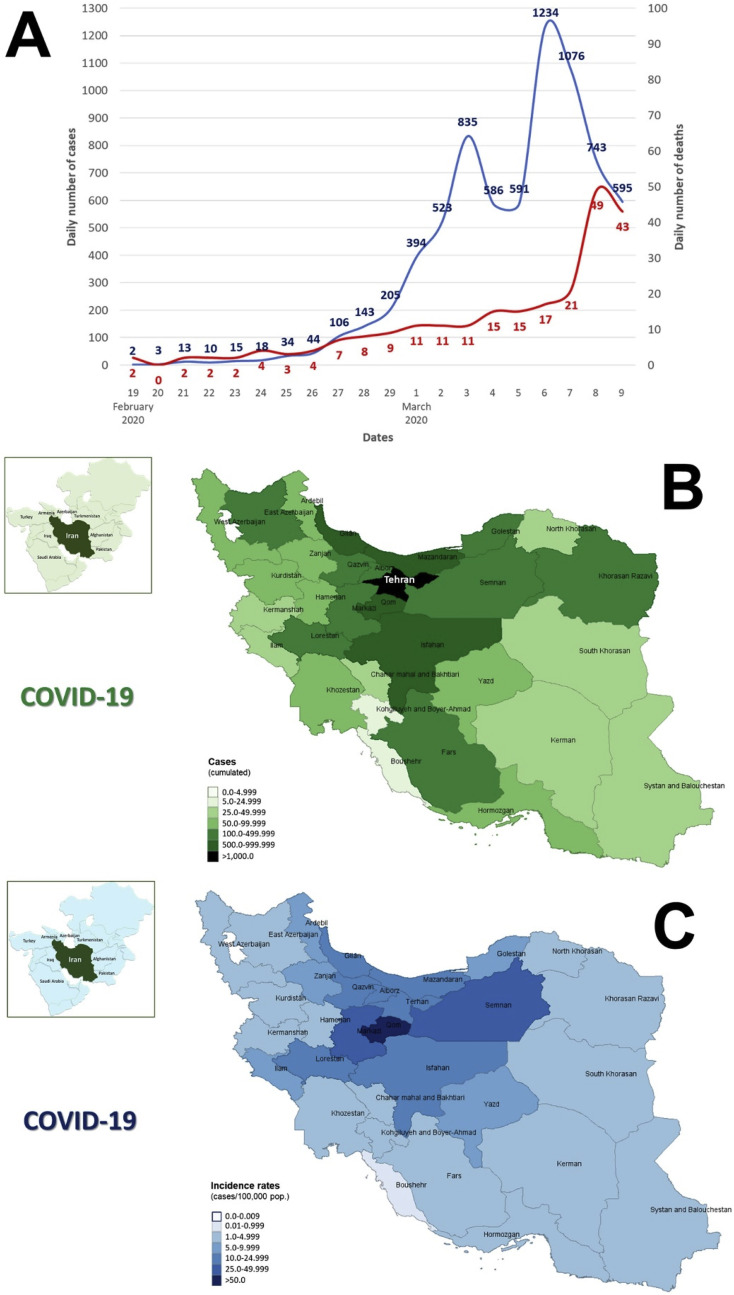 |
| --- |
| COVID-19 situation in Iran, from February 19 to March 9, 2020. A. Number of cases and deaths. B. Case distribution by the province. C. Incidence rate, case/100,000 population by the province [1]. |

**eAppendix 2**. The COREQ 32-­‐item checkllist

| No. Item | Guide questions/description | Reported on Page # |
| --- | --- | --- |
| Domain 1: Research team  and reﬂexivity |  |  |
| 1. Interviewer/facilitator | Which author/s conducted the interview? | Page # 6-7 (Setting and Study Population) |
| 2. Credentials | What were the researcher's credentials? | Page # 6-7 (Setting and Study Population) |
| 3. Occupation | What was their occupation at the time of the study? | Page # 7 (Setting and Study Population) |
| 4. Gender | Was the researcher male or female? | Page # 7 (Setting and Study Population) |
| 5. Experience and training | What experience or training did the researcher have? | Page # 7 (Setting and Study Population) |
| 6. Relationship with participants established | Was a relationship established prior to study commencement? | Page # 7-8 (Data Collection Procedure) |
| 7. Participant knowledge  of the interviewer | What did the participants know about the researcher? | Page # 10 (Ethical Considerations) |
| 8. Interviewer  characteristics | What characteristics were reported about the interviewer/facilitator? | Page # 7 (Setting and Study Population) |
| Domain 2: study design |  |  |
| 9. Methodological  orientation and Theory | What methodological orientation was stated to underpin the study? | Page # 4-12 (Methode) |
| 10. Sampling | How were the participants selected? | Page # 6 (Theoretical Sampling ) |
| 11. Method of approach | How were participants approached? | Page # 4-12 (Methode) |
| 12. Sample size | How many participants were in the study? | Page # 6 (Theoretical Sampling ) |
| 13. Non-­‐participation | How many people refused to participate or dropped out? Reasons? | NA |
| 14. Setting of data collection | Where was the data collected? | Page # 4-7 (Data Collection Procedure) |
| 15. Presence of non-­‐  participants | Was anyone else present besides the participants and researchers? | NA |
| 16. Description of sample | What are the important characteristics of the sample? | Page #; Table 1 |
| 17. Interview guide | Were questions, prompts, guides provided by the authors? | eAppendix 3. Interview Topic Guide |
| 18. Repeat interviews | Were repeat interviews carried out? | NA |
| 19. Audio/visual recording | Did the research use the audio or visual recording to collect the data? | Page # 7-8 (Data Collection Procedures) |
| 20. Field notes | Were ﬁeld notes made during and/or after the interview? | Page # 9 (Analytic and Methodological Memos and Diagrams  ) |
| 21. Duration | What was the duration of the interviews | Page # 4-12 (Data Collection Procedure) |
| 22. Data saturation | Was the data saturation discussed? | Page # 5 (Design) |
| 23. Transcripts returned | Were transcripts returned to participants for comment and/or correction? | NA |
| Domain 3: analysis and  ﬁndings |  |  |
| 24. Number of data coders | How many data coders coded the data? | Page # 9(Data Analysis) |
| 25. Description of the  coding tree | Did the authors provide a description of the coding tree? | eAppendix 4. Codebook |
| 26. Derivation of themes | Were themes identiﬁed in advance or derived from the data? | Page # 13-15 (Result) |
| 27. Software | What software, if applicable, was used to manage the data? | Page # 8 (Data Analysis and Software Program) |
| 28. Participant checking | Did participants provide feedback on the ﬁndings? | Page # 10 (Rigor) |
| 29. Quotations presented | Were participant quotations presented to illustrate the themes/ﬁndings? Was each quotation identiﬁed? | Page # 13-15 (Results) |
| 30. Data and ﬁndings  consistent | Was there consistency between the data presented and the ﬁndings? | Page # 12-18 |
| 31. Clarity of major themes | Were major themes clearly presented in the ﬁndings? | Page # 13-15 (Results) |
| 32. Clarity of minor  themes | Is there a description of diverse cases or discussion of minor themes? | Page # 9-18 |

**eAppendix 3.** The interview guide

**A. Warm-up questions**

- Tell me, how are you?
- For how long have you been referred to the thalassemia clinic?

**B. Interview questions**

1. Please tell me what you currently know about Coronavirus (COVID-19)?
 Probing question: Where you get information on COVID-19?
 Probing question: How do you verify whether the information is accurate or not?

2. What safety measures have you taken for your safety during COVID-19?

Probing question: hand washing, use of sanitizer, social distancing, covering your mouth, avoiding touching your eyes, nose, and mouth, wearing a face mask, avoiding close contact with someone who could be sick, so on.
3. Has COVID-19 affected your day-to-day life? If so, how?
 Probing question: How has COVID-19 affected your workflow?
 Probing question: How has COVID-19 affected your health?
 Probing question: How did you respond?
4. Has the healthcare system provided you with training on COVID-19?

Probing question: What kind of education and training? What was the education method?
 Probing question: Do you feel prepared after the education and training?
5. Has the healthcare system instructed you to take certain precautions for COVID-19? If so, what and how?
 Probing question: What extra precautions are you taking beyound what the healthcare system recommends? And from whom?
6. How the healthcare system understand that you have caught/have not caught COVID-19?
7. Have you talked to other patients about the experiences of COVID-19?
 Probing question: Have you talked about education on COVID-19? Have you talked about precautions and the best practices?

Probing question: If you could speak with a peer about the experiences with COVID-19, what would you like to know or talk about?
8. How your family supports you during the COVID-19 pandemic?

Probing question: Do you receive support from your parents or your siblings?

Probing question: Did they play these roles in your life before the pandemic?
9. Have you encountered a patient with COVID-19 or a suspected patient?
 Probing question: If so, will you please describe your experience?

Probing question: What makes you worry the most when encountering a thalassemia patient with COVID-19?
10. Have you had the common symptoms of COVID-19 including shortness of breath, fever, cough, etc.?
 Probing question: If so, what did you do?
11. What does make you feel worries about the current coronavirus situation in terms of job or income?
 Probing question: Have you lost your job or have you changed it during the COVID- 19 pandemic?

12. What does make you feel worried about the current coronavirus situation regarding your access to the healthcare system?

Probing question: Have you lost/changed your appointment during the COVID- 19 pandemic? If so, will you please describe that experience?

13. How did you feel during the COVID-19 pandemic?

Probing question: What was your main form of stress during the COVID-19 pandemic?
 Probing question: What would make you feel more comfortable during the coronavirus situation?

Probing question: What did you feel or think about this health issue?

Probing question: How do you cope with anxiety and fear related to the COVID-19 pandemic?

Probing question: Do you feel the need to have mental health programs or other measures to overcome anxiety, fear, and stress in this pandemic situation?

14. Do you think that novel coronavirus will impose serious damages to your community, if adequate safety measures are not taken?

15. Do you think you can protect yourself against coronavirus?

16. What changes have happend in the healthcare system during COVID-19 pandemic?

Probing question: How have these changes affected your treatment?

Probing question: What do you wish the healthcare system to do as the matter of support?
17. Are there additional concerns or issues you would like to talk about? Do you have any questions
from me?

**eAppendix 4.** The codebook

| Themes and subthemes developed in this study about the experiences of Iranian patients with beta-thalassemia major during the COVID-19 pandemic | | |
| --- | --- | --- |
| Themes | **Subthemes** | **Open codes** |
| Changing physical health | Somatic sensation of physical symptoms | - Pain - Weakness - Fatigue and lack of energy - Loss of appetite - Shortness of breath - Trouble with meeting essential needs - Compultion to spend time in bed |
|  | Adverse effects of postponement of treatments | - Low hemoglobin - Iron overload - Recurrence of cardiac events - Borderline blood sugar - Increased comorbidities |
| Emotional and psychological reactions | Negative emotions in the early stage | - Fear of blood transfusion - Fear of viral infection - Anxiety caused by the lack of knowledge - Anxiety caused by environmental changes - Fear from the strange appearance of healthcare workers - Fear of not being recovered from the covid-19 due to comorbidities - Fear from healthcare workers baceaus of being infected - Concern provoked by the scarcity of the seasonal influenza vaccine |
|  | Concerns provoked in each peak | - Being worried about the reduction of blood donation - Anxiety due to facilities cancellation policies in NGOs - Concern provoked by the inaccessibility of healthcare facilities - Concern over the anticipated lack of thalassemia support by the government |
|  | Depression | - Feeling unhappy - Feeling unmotivated - Feeling not reaching goals - Changes in sleep patterns - Depression caused by the cancelation of entertainment programs |
|  | Affective context of trust | - Trust in God in all circumstances - Trust in self-prevention abilities - Trust in the healthcare environment - Lack of trust in media - Trust in healthcare workers - Lack of trust in organizations and the government |
|  | The gradual emergence of positive emotions | - Calmness after notifying the recovery of infected patients - Calmness after seasonal influenza vaccination - Satisfaction from multiple social support sources |
| Functionality and adaptation to new realities | Planning and safety | - Adherence to infection prevention and control - Safety and individual preparedness - Plan for preparation |
|  | Awareness and sensitization | - Attaining information effectively - Curiosity and sensitivity - Misconceptions regarding COVID-19 - Threat perceived risk - Feeling and understanding the risk - Temporary attention to the risk of COVID-19 |
|  | Use of telemedicine and virtual counseling | - Use of online psychology counseling - Use of cardiac telerehabilitation |
|  | Request for a home visit | - Home-visit nursing services - Home-visit doctor services |
|  | Putting off routine medical appointments | - Deferral blood transfusion - Preferred annually screenings instead of sequential - Reduction of refereral to the hospital to evaluate disease complications |
|  | Transitional care from hospitals/ outpatient settings to the physician office | - Visiting a doctor's office instead of a hospital outpatient clinic - Performing echocardiography in the doctor's office instead of the hospital outpatient clinics |
|  | Changes in medication adherence | - Reduced adherence to medications caused by a lack of timely preparation of medicines - Improved adherence to medications due to taking an advantage of opportunities during quarantine - Non-adherence to medications due to sleep - Changes in the life pattern |
|  | Changes in the work capacity and economical condition | - Loss of work and income - Cost of physician-led home visit care - Cost of visiting a doctor's office - Cost of purchasing face masks and disinfectants - Establishing a home-based business - Working shorter than healthy colleagues |
| Changing the nature of relationships and the scope of social support | The increasing involvement of the family in patient care | - Provision of medication by the family during COVID-19 - Preventing the patient from leaving home by the family at peaks - Making an appointment for a doctor by the family - Getting more family support rather than before pandemic |
|  | Accelerated changes in pivotal strategies by the thalassemia association | - Distribution of livelihood packages including rice, meat, and warm clothes - Delivering remote mental health services - E-Learning for patient education about the safety of blood - Cancelation of exercise and entertainment programs - Providing telephone/online follow-up on patients’ requests - Distribution of healthcare packages including masks, and disinfection gels |
|  | Loss of social connectedness | - Loss of social connectedness - Lack of the personal engagement - Avoiding social contact by others - Wasting time in social media |
|  | Social stigma associated with COVID-19 | - Concealment of COVID-19 - Social rejection caused because of being infected by COVID-19 |
| Metamorphosis of ongoing healthcare | Inappropriate interactions of the healthcare team with patients during COVID-19 pandemic | - Hidden communication behind masks and gloves - Keeping distance by nurses from patients - Unkind interaction style by the healthcare team |
|  | Slow healthcare procedures | - Personal distancing between patients - Diagnostic procedure - Disinfection after each visit - Triage pre-entry |
|  | Significant attention to‎ the protection of the patient | - Replament of doctors and changes in the treatment process - Evaluating patients for COVID-19 symptoms during the monthly visit - Equipping the thalassemia clinic with echocardiography set to prevent referal to the hospital - Free vaccination of all patients with seasonal influenza vaccine - Monitoring the implementaion of the protocol for COVID-19 - Equipping the thalassemia clinic with an isolation room - Starting specific training for COVID-19 - Avoiding patient companions to enter the ward - Replacement of face-to-face activities with making phone calls |
|  | Decreased access to the healthcare system | - Lack of access to advanced imaging for thalassemia patients - High transmission of COVID-19 to healthcare staff - Inactivity of emergency departments at least during COVID-19 peaks - Limiting outpatients' services to urgent cases - Changes in transitional care within healthcare facilities - Reduction in the number of patients evaluated per day in the doctor's office |
|  | Transfere of resources from existing programs to COVID-19 | - Laboratory and other diagnostics measures - Protective equipment - Healthcare workers - Financial suport - Medications and consumables |

NGOs: Non-governmental organizations; COVID-19: Coronavirus disease-2019

**eAppendix 5.** The code cloud

| 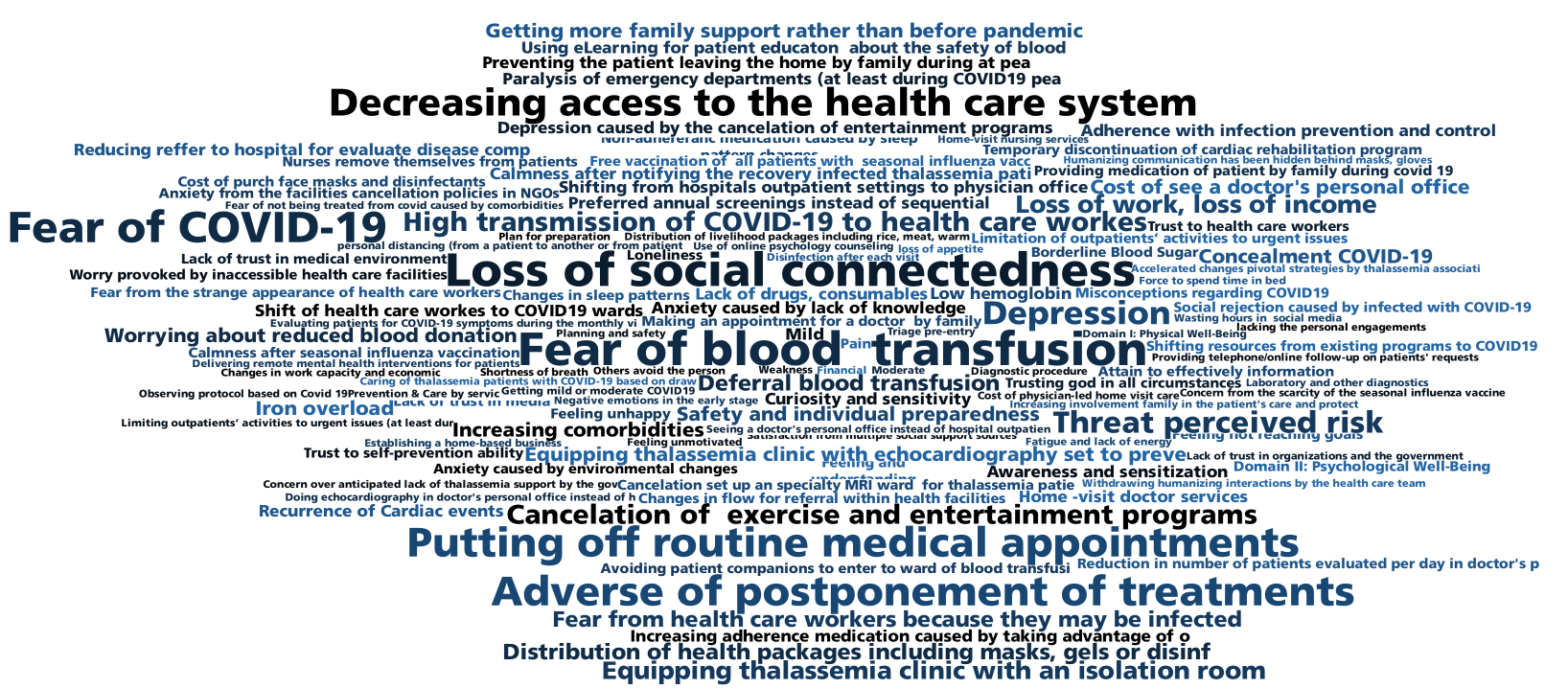 |
| --- |
| **The code cloud** |
