# Supplementary figures and images for: Understanding the impact of COVID-19 pandemic on health-related quality of life amongst Iranian patients with beta thalassemia major: a grounded theory
Source: Prim Health Care Res Dev. 2021 Nov 10;22:e67. doi: 10.1017/S146342362100013X (PMC8581459; doi:10.1017/S146342362100013X)

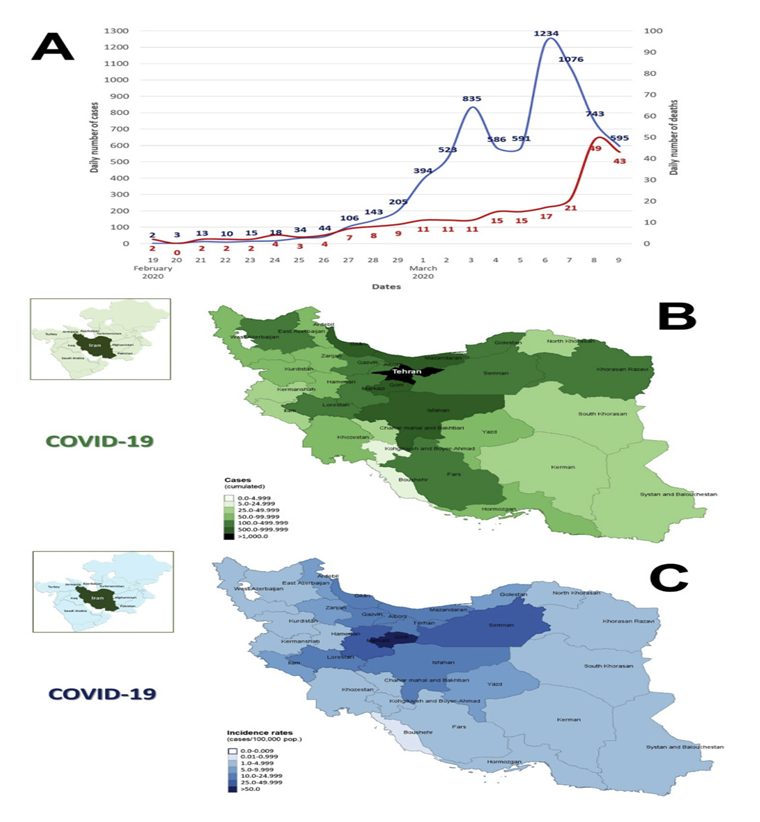

Supplement: Supplementary file 1 [file phcsup.zip › S146342362100013Xsup001/S146342362100013Xsup001.tif]

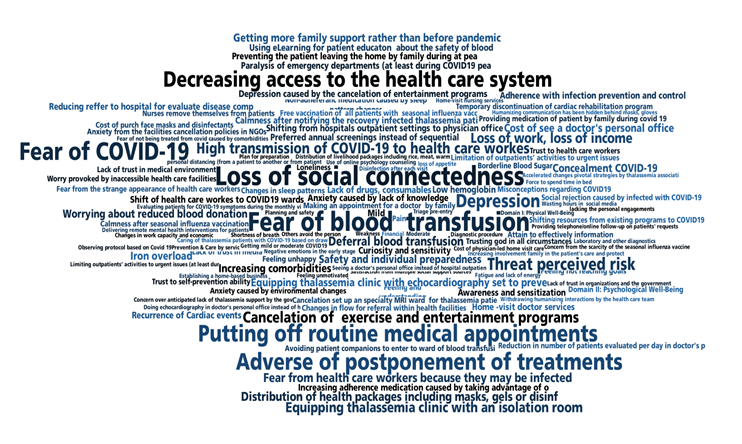

Supplement: Supplementary file 1 [file phcsup.zip › S146342362100013Xsup001/S146342362100013Xsup002.tif]
